# Using social network analysis to examine alcohol use among adults: A systematic review

**DOI:** 10.1371/journal.pone.0221360

**Published:** 2019-08-22

**Authors:** Justin Knox, John Schneider, Emily Greene, Joey Nicholson, Deborah Hasin, Theo Sandfort

**Affiliations:** 1 Department of Epidemiology, Columbia University Mailman School of Public Health, New York, NY, United States of America; 2 Pritzker School of Medicine, University of Chicago, Chicago, IL, United States of America; 3 Chicago Center for HIV Elimination, University of Chicago, Chicago, IL, United States of America; 4 Department of Medicine, University of Chicago, Chicago, IL, United States of America; 5 Health Sciences Library, New York University School of Medicine, New York, New York United States of America; 6 Department of Psychiatry, NYS Psychiatric Institute and Columbia University, New York, New York United States of America; 7 HIV Center for Clinical and Behavioral Studies, NYS Psychiatric Institute and Columbia University, New York, New York United States of America; Cinvestav-Merida, MEXICO

## Abstract

**Background:**

Alcohol use and abuse constitute a major public health problem and identifying their determinants is a priority. Social network analysis can indicate how characteristics of social networks are related to individual health behaviors. A growing number of studies have used social network analysis to examine how social network characteristics influence adult alcohol consumption, but this literature has never been systematically reviewed and summarized. The current paper systematically reviews empirical studies that used social network analysis to assess the influence of social network characteristics on drinking behaviors in adults.

**Methods:**

A literature search of PubMed/MEDLINE, EMBASE, PsycINFO and Web of Science databases and a review of the reference lists of retrieved articles was conducted in March 2019. Two reviewers independently screened 5,510 non-duplicate records, and further screened the full text of 150 articles to determine their eligibility for inclusion. Seventeen articles were judged eligible and included.

**Results:**

Most studies were conducted among young adults (mean age<30), in university settings or follow up visits with adolescent networks moving into adulthood. The objectives and methods of the included studies were heterogeneous. All included studies reported a statistically significant association between a social network characteristic and an alcohol consumption-related outcome. Social network members drinking behaviors were associated with participants’ drinking behaviors in multiple ways.

**Discussion:**

In young adults, among whom the majority of identified studies were conducted, with whom they socialize and how they socialize appears to be associated with alcohol consumption; this was observed across methodologies and settings. We still know very little about the relationship of social networks to drinking in older age groups, and in populations most impacted by alcohol. As social networks appear to play a role in the consumption of alcohol in young adulthood, interventions that utilize social networks to help reduce harmful alcohol consumption should be considered.

## Introduction

Alcohol consumption is prevalent worldwide, with more than 2.4 billion people (33% of the global population) being current drinkers [1]. In the US, the prevalence of two forms of excessive alcohol consumption, high-risk drinking and alcohol use disorder, have increased substantially in adults over the past decade, such that 1 in every 8 adults report past-year high-risk drinking [2] and the prevalence of lifetime alcohol use disorder is high [3]. Alcohol use is also a leading cause of global disease burden and health loss [1]. Risk of all-cause mortality is positively associated with level of alcohol consumption, such that any level of consumption is potentially harmful [1]. These recent findings are consistent with the well-demonstrated relationship of excessive alcohol consumption to numerous adverse health consequences [2–5], and to increased morbidity and mortality worldwide [6–8]. Excessive alcohol consumption additionally places psychological and financial burdens not only on those who engage in these behaviors, but also their families, friends, coworkers and society as a whole [9, 10].

Many studies have identified individual-level determinants of alcohol consumption [2, 3, 11], but. these studies have limited their focus to psychological or other individual characteristics of alcohol users. Socio-ecological models [12] point to larger social units, ranging from networks to institutional factors, as potential drivers of alcohol use. In this regard, one step beyond looking at individual risk factors is to consider the influence of social networks on alcohol-related outcomes. Social network analysis can be used to show how peer drinking behavior and patterns of relationships that connect social actors influence an individuals drinking behavior. That alcohol consumption can both influence choice of relationships (e.g. selecting drinking buddies as friends) and be influenced by them (e.g. being pressured by peers to drink alcohol), suggests that this is an area ripe for investigation.

Social network analysis is the term applied to a set of theories and methods used to study social interactions between individuals, and how these social interactions influence various outcomes [13]. A fundamental tenet in social network analysis is that it incorporates information about relationships between members of a shared social network. The most comprehensive approach that researchers use to collect data on social networks is termed a *sociometric* network approach, which involves interviewing multiple members (ideally all members) of a social network [13]. When a social network of interest is complete (i.e. bounded), rosters can be used to facilitate selecting the sampling frame and identifying connections between social network members. Alternatively, social network data can be collected by interviewing an individual (i.e. an index) and then interviewing the social network members that an individual nominates (i.e. alters). This process can be carried out successively, for as many waves as are needed until saturation of network members and the ties between them are achieved [14]. In the case that a complete network is not sampled, collecting data among multiple members of a social network at least allows for the creation of directed graphs, where there can be directionality in the relationships among members of a shared social network. These approaches allow one to measure the actual behavior of shared social network members, rather than just an individual’s perception of shared social network members’ behavior, which is known to be differentially biased (i.e. they tend to reflect the behaviors of the individuals describing them) [13, 15]. Social network data, both *sociometric* and that limited to directed graphs, provide a global view of a social network and its structure, including multiple members’ perspectives, and thus they have great analytic possibilities. This approach has been effectively used in schools, for example, as networks are characterized at the classroom level and diffusion of behaviors measured and intervened upon [16–18].

The relevance of social networks to communicable diseases that require the spread of pathogens between people is obvious [19–25]. Social networks have also been shown to influence non-communicable diseases [26, 27], and health-related behaviors [28–35]; both unhealthy behaviors, such as drug use and specific HIV related behaviors [36–38], and healthy behaviors, such as smoking cessation or HIV prevention [39–43]. Specific interventions have also been developed that utilize social networks to promote behavior change [44].One way that social networks have been shown to influence the health of their members is that the characteristics of the people in one’s social network provide a context for one’s own behavior and norms. Members of a shared social network might influence each other through persuasion, sharing information or expressing support. In order to study these peer effects, the characteristics of social networks members can be measured to assess their relationships to the characteristics of other members in a shared social network (e.g., is an individual’s alcohol use associated with the alcohol use of one’s friends). [45–48]. Another way to look at peer effects is by looking at the distribution of a characteristic throughout a social network; if a characteristic is not randomly distributed then there is said to be clustering, and a network is said to be homophilous on that characteristic. Homophily can either be a result of confounding (peers are similar because of a shared environment), selection (i.e. individuals become friends with others who are like them), or socialization/induction (friends influence each other to become more similar). Selection and socialization are the mechanisms for peer influence; attempting to identify and isolate their effects is the theoretical and empirical basis for using social network analysis.

Social network structure has also been found to be an important driver of health behaviors [49, 50]. These structures can include network positions (central, bridging), sub-network groupings (ie. faction, clique) and cohesion among others [51]. For example, social network ties (i.e. relationships), in themselves, can be considered as potential determinants of health (e.g. is a member of a social network well connected or fairly isolated) [52–54]. Also, the total size of one’s social network can matter or whether a network is densely or loosely connected. An individual’s position within a network can also matter, as it might reflect their level of prestige within that group or they play a critical role in terms of transmission. These means of influence can be inter-related; as for example, one’s degree of connectedness to a specific social network is positively associated with one’s likelihood of reflecting the normative behavior of that group [52].

Empirical research studies have used social network analysis to examine alcohol-related outcomes, although these have mostly been conducted among adolescent populations [28–35, 55, 56]. A systematic review on the use of social network analyses to understand risky behaviors focused solely on studies of adolescents [57], and only included studies that used data from the National Longitudinal Study of Adolescent to Adult Health (Add Health) [58]. This review identified eight studies that looked at alcohol-related outcomes, all of which found that adolescent friendship networks promoted drinking alcohol [57]. Various types of friendships were found to influence adolescents’ drinking behavior, with friendships that mattered (based on levels of closeness or by being reciprocated), being more likely to exert influence. The included studies that used longitudinal data showed that individuals who have friends who drink or are linked to friendship networks where individuals drink are at increased risk for drinking themselves, initially and over time.

How social networks influence drinking behaviors could be substantially different among adults compared to adolescents [59]. For example, peer groups tend to expand and diversify from adolescence into adulthood [60]. Not only does one’s peer group change, but so does the amount of time one spends with peers and how one interacts with them [61]. Perhaps, as a result, the influence of peers is believed to wane in later adulthood [62]. Looking at things beyond homophily, the impact of social network structure on alcohol might also be qualitatively different in adults compared to adolescents. For example, centrality is the most common social network analysis measure used in alcohol research among adolescents, and we know that more popular adolescents (i.e., those with more friends) have higher levels of alcohol use [57]. However, in adulthood, having more friends may lose its association with alcohol use as it becomes less important in reflecting perceived social norms. Also, centrality may operate differently in adolescents and in adults above the legal drinking age as such adults would not need to rely on peers to access alcohol. Individual studies have examined the relationship of social networks to alcohol use among adults. However, thus far, this literature has not yet been summarized in a systematic review. The lack of a review on this topic in adults represents an important gap in knowledge. The current paper aims to fill this gap by systematically identifying and describing empirical studies that used social network analysis to evaluate alcohol-related outcomes among adults (persons who are 18 years and older). We then synthesize the findings of the identified studies that statistically measured the influences of social network characteristics on alcohol use in adults.

## Methods

This review was informed by the Preferred Reporting Items for Systematic Reviews and Meta-Analyses (PRISMA) Statement. The PRISMA checklist [63, 64] is shown in S1 Table.

### Data sources and literature search

A literature search of 4 databases (PubMed/MEDLINE, EMBASE, PsycINFO and Web of Science, Sociological) was conducted in March 2019 to identify studies that used social network analysis to evaluate the effects of social network characteristics on alcohol use. Keyword and terms used in search strategies varied based on the database to account for distinct indexing criteria and are described in detail in S2 Table. To identify additional studies not found in the literature search, the reference lists of relevant review articles of social network analysis [13, 44, 57] and included articles were reviewed. The literature search was conducted with guidance from an Education and Curriculum Librarian who serves as the Coordinator for Systematic Review Services at the New York University School of Medicine.

### Screening

Duplicates were removed and screening of retrieved articles by title and abstract was conducted by two independent reviewers using Covidence systematic review software (Veritas Health Innovation, Melbourne, Australia. Available at www.covidence.org). Final inclusion was determined by two independent reviewers (JK & EG) screening the full-text of potentially eligible articles using the following criteria. Studies were eligible to be included in the review if they (1) were published in a peer-reviewed journal, (2) were written in English, (3) were conducted in human populations, (4) utilized a social network analysis design that included directed graphs (where there is directionality in the ties) and which data were collected beyond dyadic pairs (i.e., data linking participants were measured or inferred (e.g., known roommates were considered to be connected) and data were collected among alters for 3 or more connected individuals) and network measures were calculated based on this data, (5) evaluated an alcohol-related outcome, and 6) included a majority of participants that were adults (18 years or older). Studies were excluded if (1) the impact of social networks was assessed using simulations, (2) the study described qualitative research or was a case study. In the few cases on which opinions about inclusion differed, the reviewers met and reached consensus through discussion.

### Quality assessment

We used a 12-item quality assessment tool (S3 Table) to evaluate study relevance and methodology. This tool was developed using modified sets of criteria from other quality assessment tools for assessing observational studies [65] and network studies [66]. The tool assessed description of: the research question, data collection procedures (data source(s), study setting, sample size, response rate, sample selection), measures used (exposure and outcomes), analysis of social network data, results and findings, strengths and limitations, and conclusions drawn.

### Data extraction and synthesis

A data extraction form was developed to extract information on study objectives, study design and sampling approach, data collection method, setting and target population, participants, social network data collection procedures, outcome measurement, social network analysis methods used to analyze the data and calculated measures, statistical analyses used (as relevant to social network analysis), and key findings.

## Results

### Identifying empirical studies

**Fig 1:**
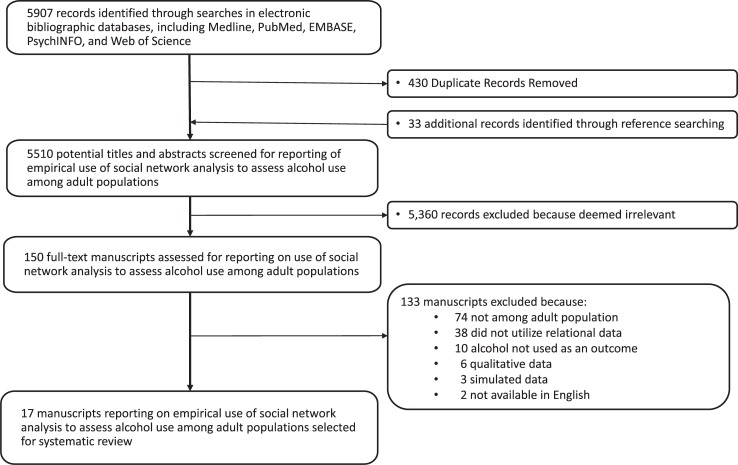
Flow diagram of search strategy and selection process.

The literature search yielded 5,907 records for screening (5,477 after removing duplicates), and an additional 33 were included based on reference list review for a total of 5,510. The full text of 150 articles was reviewed to determine eligibility and 17 articles were judged potentially eligible and further assessed for quality and relevance (S4 Table). Scores on the quality assessment tool scale ranged from 78%-100% (mean = 95%). Based on these scores, all 17 articles were included in the review (Fig 1) [67–83]. Because the methodologies used in the included studies were heterogeneous, they did not lend themselves to meta-analysis. Therefore, a narrative synthesis of the studies will be conducted.

### Population and setting

Among the included studies, 13 were conducted in the United States [67–70, 72, 73, 75, 76, 78, 79, 81–83], 3 were conducted in Europe [71, 77, 80] and one was conducted in Africa [74]. Most studies (n = 15) were conducted among young adults [67–74, 76–81, 83], the majority (n = 12) of which were among university students [67–71, 73, 76–81]. Five studies were conducted in community settings [72, 74, 75, 82, 83]. Mean age of the participants ranged from 18 years to 51 years. Proportion of male participants ranged from 25% [81] to 100% [71, 74]. Characteristics of the included studies are summarized in Table 1.

**Table 1:** Summary of characteristics of n = 17 social network studies included in the systematic review.

Characteristic	n (%)
Country	
US	13 (76)
Europe (Germany, Belgium, Netherlands)	3 (18)
Africa (South Africa)	1 (6)
Setting	
University	12 (71)
Community	5 (29)

### Sampling and study design

Sample selection procedures included the use of respondent-driven sampling, recruitment of peer groups, and the collection of complete sociometric social networks. The studies that used more robust sociometric social network sampling procedures [67–72, 77, 78, 81, 82] either collected data on complete (i.e. bounded) networks [77, 81] or used complete rosters but limited the number of peers that an individual was able to nominate [67–69, 71]. Two of the studies relied on peer nominations or recruitment without the availability of rosters [72, 74, 82]. Sample sizes ranged from 34 [81] to 12,067 [82]. Of the studies, 11 used cross-sectional data [67–70, 72–74, 77–80, 83], 5 used a longitudinal study design [71, 75, 76, 81, 82], and one was a naturalistic observation study in a ‘bar-lab’ [80]. Details of the individual studies, including objectives, study period and design, setting, participant characteristics, data sources, social network measures, analytic methods, and major findings are presented in Table 2.

**Table 2:** Summary of n = 17 social network analysis studies with alcohol as an outcome among adult populations.

Study	Objective	Study details^1^	Study design	Data sources	Social network measure(s)	Statistical analyses^2^	Major findings related to the social network analyses
Barnett et al. (2014a) [67]	Investigate five different social network characteristics (indegree centrality, betweenness centrality, outdegree, indegree reciprocity, and outdegree reciprocity) for alcohol use and alcohol-related problems in a college residence network	US; 129 students living on a college campus in the NE; 48% male	Cross-sectional	Interview with SNQ of up to 10 people who lived in the residence hall	Indegree centrality, betweenness centrality, outdegree, indegree reciprocity, outdegree reciprocity	Simultaneous autoregressive (SAR) autocorrelation models	Two network characteristics were significantly associated with alcohol use and a third showed an association for women only. Outdegree was significantly positively related to number of heavy drinking days. Betweenness centrality was significantly positively related to number of alcohol problems. Betweenness centrality and indegree reciprocity were significantly associated with greater alcohol problems for women.
Barnett et al. (2014b) [68]	Use a college residence hall peer network to examine associations between peer behaviors and alcohol use, marijuana use, and exercise behavior	US; 129 students living on a college campus in the NE; 48% male	Cross-sectional	Interview with SNQ of up to 10 people who lived in the residence hall	Cluster identification based on betweenness, weekly volume of alcohol consumed by direct ties	Network autocorrelation models	Community detection cluster analysis used only directed ties to detect subcommunities of individuals, and the comparison of those groups established that they differed significantly on demographic, activity, and behavior profiles, including alcohol use and alcohol-related problems. The drinking volume of nominated peers was significantly positively associated with participant drinking volume.
DiGuiseppi et al. (2018a) [69]	Investigate the association between actual and perceived peer drinking and participant drinking, and the possible moderating effect of resistance to peer influence	US; 1342 students enrolled in their first semester at a mid-sized, private university in the NE; 18.7 years = mean age; 45% male	Cross-sectional	All students in the class were included in the social network, participants were asked to select their social network connections from a list of all students.	Binge drinking frequency of important peers	Two separate network autocorrelation models were conducted, one for perceived peer drinking and one for actual peer drinking	Participant's binge drinking frequency was positively associated with both perceived and actual norms. Resistance to peer influence weakened the effect of perceived peer binge drinking on participant binge drinking, but did not interact with actual norms.
DiGuiseppi et al. (2018b) [70]	Investigate the association between social network characteristics, alcohol use, and alcohol-related consequences among first-year college students at one university	US; 1342 students enrolled in their first semester at a mid-sized, private university in the NE; 18.7 years = mean age; 45% male	Cross-sectional	All students in the class were included in the social network, participants were asked to select their social network connections from a list of all students.	Indegree (popularity), outdegree (expansiveness), reciprocity, density (the proportion of completed triads, out of all possible triads, among participants’ peer nominations), binge drinking norms (average binge drinking frequency among all of the peers that participants nominated)	Four network autocorrelation models were conducted, using the following outcome variables: (1) average number of drinks per week, (2) heavy drinking frequency, (3) alcohol-related consequences, and (4) alcohol-related consequences after controlling for drinks per week.	Popularity (i.e., indegree) and descriptive norms showed significant positive associations with average number of drinks per week,heavy drinking frequency, and alcohol-related consequences and remained significantly associated with alcohol-related consequences even after controlling for alcohol consumption.
Giese et al. (2017) [71]	Explore the role of friendship reciprocity in shaping frequency and quantity of alcohol consumption among university Freshmen	Germany; 57 first semester psychology students at the University of Konstanz from 2008–2009; 20.9 years = mean age (at baseline); 25% male	Longitudinal	Interview with SNQ that asked participants to nominate the 3 people that they liked most that week from the full list of participants	Outdegree nominations and indegree nominations	Multilevel regression models	Participants’ frequency of drinking was associated with reciprocating friends’ frequency of drinking. Participants’ quantity of drinking was associated with friends’ quantity of drinking regardless of reciprocation.
Janulis et al. (2015) [72]	Examine relationships between network (i.e., transitivity and network size), dyadic (e.g., age difference), and individual characteristics and drug and alcohol behavior with substance use alters to better understand the social and contextual factors associated with substance use behavior among young MSM	US; 156 young MSM; 20.1 years = mean age (at baseline); 100% male	Cross-sectional	Individual interviews and RDS recruitment data	Transitivity, network size, dyadic frequency and type of drug use	Logistic mixed models with random intercepts	A participant’s drug use and a participant’s frequency of drug and alcohol use with substance use alters were positively associated with the network transitivity of their substance use network. Thus, the ties between alters that an individual uses substances with is related to the type and frequency of substance use with those alters.
Kenney et al. (2017) [73]	Examined how misperceptions of residence hall peers, both overall using a global question and those designated as important peers using person-specific questions, were related to students’ personal drinking behaviors	US; 108 students living on a college campus in the NE; 49% male	Cross-sectional	Interview with SNQ of up to 10 people who lived in the residence hall	Self-reported and peer-reported alcohol consumption	Network autocorrelation models	Participants accurately perceived the drinking of nominated friends but overestimated the drinking of residential peers. Misperceptions of peer drinking predicted personal drinking behavior.
Knox et al. (2017) [74]	describe alcohol use among black South African MSM and identify determinants that put them at risk for hazardous drinking	South Africa; 480 MSM living in Pretoria and the surrounding townships; 24 years = mean age; 100% male	Cross-sectional	Individual interviews and RDS recruitment data	outdegree centrality, proportion of a participant’s ties that screened positive as hazardous drinkers using the AUDIT-C	Multivariable logistic regression	Men whose social networks included a higher proportion of hazardous drinkers were more likely to be hazardous drinkers themselves.
Latkin et al. (1996) [75]	Examine the prospective association between baseline self-reported drug and alcohol use of the network members of injection drug users, and self-reported sexual behaviors and alcohol use at 5-month follow-up	US; 71 nontreatment inner-city injection drug users who volunteered for a network-oriented HIV preventive intervention and 227 members of their drug networks from 1991–1992; 38 years = mean age; 85% male	Longitudinal	Detailed, face-to-face interview on background, HIV-related behaviors in the prior 6 months, and SNQ where they were required to provide names and descriptive information on their network members. Indexes were compensated $25 for each drug-sharing network member that came in to be interviewed.	Drug networks’ mean baseline level of alcohol consumption	Prospective multiple logistic regression	Drug networks’ mean baseline level of alcohol consumption was a significant predictor of indexes’ daily alcohol consumption in the prior six months.
Lau et al. (1990) [76]	Explore sources of stability and change in young adults' beliefs and behavior concerning drinking during the first 3 years of college	US; 947 students admitted to Carnegie Mellon University and their parents; 69% male; 18 years = mean age (at baseline)	Longitudinal	Interviews among participants, their parents and up to 2 other participants in the study- roommates and people named by the youths as their best friends at college.	Parents’ alcohol beliefs, parents’ alcohol consumption, peers’ alcohol beliefs, peers’ alcohol consumption,	Structural equations analysis with latent variables	Parental influence on their children's drinking beliefs and drinking behavior are present at baseline and persist, despite weakening, at least through the college years. Peers drinking behavior was associated with participant’s drinking behavior.
Lorant et al. (2015) [77]	Analyze the role of peers and of social position within a university network in drinking behavior	Belgium; 487 undergraduates in 2 faculties (Engineering and Psychology) in a university in 2010; 45% male	Cross-sectional	Paper-pencil questionnaires with SNQ where participants were provided with a complete list of all students to identify those with whom they had the following relationships: friends, roommates, studying or working with, and spending leisure time with.	In-degree, closeness, cross-gender relationships, effective size	Poisson regression with permutation tests to assess the distribution of the estimates.	Being socially close to binge drinkers was associated with a higher frequency of binge drinking; higher for reciprocated ties than non-reciprocated. The risk of binge drinking increased with centrality but decreased with social capital. Having cross-gender relationships decreased the risk of binge drinking. The effect of centrality and gender on binge drinking depends on the composition of the network.
Meisel et al. (2018) [78]	Investigate the network of social connections between drinkers on their heaviest drinking occasions	US; 972 students enrolled in their first semester at a mid-sized, private university in the NE who reported past-month drinking; 18.7 years = mean age; 45% male	Cross-sectional	All students in the class were included in the social network, participants were asked to select their social network connections from a list of all students. Participants who self-reported drinking in the past 30 days were additionally asked to indicate which of the people named in their sociocentric network were there.	Maximum drinking day: indegree, outdegree, betweenness centrality, mutuality, and ego density	Network autocorrelation models were conducted to examine if network indices were associated with the participant's maximum number of drinks.	The total number of times a participant was nominated as being present on another students' heaviest drinking occasion (i.e., maximum drinking day indegree) and the number of drinks consumed by the participant's nominated ties on the ties' maximum drinking days both independently were associated with a participant's maximum number of drinks.
Ott et al. (2016) [79]	Learn about the unknown average number of alcoholic drinks consumed on drinking days and the association between certain personal characteristics and alcohol consumption	US; 125 students living on a college campus in the NE who nominated other network members or who were nominated by other network members; 47% male	Cross-sectional	Interview with SNQ of up to 10 people who lived in the residence hall	Self-reported and peer-reported alcohol consumption	Novel Bayesian comparative calibration model that uses covariate information to characterize the joint distribution of both self and peer-reports on the network for estimating discrepancies in network surveys, then applied to the data for full Bayesian inference.	Use of peer-reports improves estimates of self-reported alcohol consumption. Peer-reports of alcohol consumption are overestimates. Men tended to have larger discrepancies than women.
Overbeek et al. (2010) [80]	Assess the relative importance of best friends’ alcohol use versus general levels of alcohol use in the peer setting for predicting young adults’ alcohol use	Netherlands; 221 young adults in 28 peer groups; 46% male majority groups	Naturalistic observation study	10-minute questionnaire followed by 2 hours observed drinking in a bar-lab	Peers’ quantity of alcohol consumption during the observation period	Multilevel regression analysis using both fixed and random effects	Average peer group levels of alcohol consumption was the strongest predictor of youths’ alcohol consumption in an experimental setting. This finding was less pronounced for females.
Phua (2011) [81]	Examine the influence of popularity and conforming to perceived peer norms on smoking and drinking among college fraternity members using social network analysis	US; college fraternity at private university in SW; 34 freshmen pledges; 20.1 years = mean age (at time period 1); 100% male	Longitudinal	Interview with SNQ of other fraternity members	Homophil, popularity (indegree nominations)	ANOVA density models; Quadratic Assignment Procedure correlation analyses	The network became more homophilous with regards to drinking. Popularity in the fraternity network significantly predicts heavier drinking (i.e. he more popular a member the more likely he is to be a heavier drinker)
Rosenquist et al. (2010) [82]	Explore quantitatively whether alcohol consumption behavior spreads from person to person in a large social network of friends, coworkers, siblings, spouses, and neighbors, followed for 32 years.	US; The Framingham Heart Study; 12,067 persons assessed at several time points between 1971–2003; 50.9 years = mean age; 48% male	Longitudinal	Participant data, collected every 2 to 4 years, includes physical examinations, laboratory tests, noninvasive cardiac and vascular testing, battery testing. questionnaire results, demographic information, and SNQ self-described social ties, collected in each of the 7 waves of the study.	Alcohol consumption of social network ties at various degrees of separation. Clustering in alcohol consumption (homophily, confounding, induction)	Longitudinal logistic regression models using GEE to account for multiple observations. Observed clustering of alcohol consumption within the network compared with 1000 simulated networks with same topology and prevalence of drinking as the observed network, but with the incidence of drinking randomly distributed across members.	Participants are 50% more likely to drink heavily if a person they are directly connected drinks heavily. The size of the effect is 36% for people at 2 degrees of separation and 15% for people at 3 degrees of separation. The effect disappears at 4 degrees of separation. Each heavy drinker in a participant’s social network increased the likelihood of drinking heavily by 18% and decreased the likelihood of abstinence by 7% but had no effect on moderate alcohol consumption behavior. Female contacts are significantly more likely than male contacts to influence the spread of heavy alcohol consumption.
Tucker et al. (2015) [83]	Investigated whether substance use among emerging adults living in disadvantaged urban areas was influenced by peer and family social network messages that variously encouraged and discouraged substance use.	US, Birmingham, Alabama; 344 residents of lower income neighborhoods recruited via RDS; 18.9 years = mean age; 68% female	Cross-sectional	Individual 1.5-hour interviews and RDS recruitment data	Peer substance users in participants’ immediate social networks	Linear regression	Substance use (alcohol and other drugs) by close network members was associated with global substance involvement but not alcohol involvement, specifically.

^1^Participant age and sex and study dates are included if it was reported in the article

^2^Includes statistical tests that specifically incorporated network measures

Abbreviations

SNA = social network analysis; SNQ = social network questionnaire; RDS = Respondent driven sampling; MSM = men who have sex with men

Definitions

Nodes: Distinct members of a social network (e.g., study participants)

Ego: An individual focal node providing information about their social network

Alter: The nodes to whom an ego is directly connected

Ties (edges): Representations of relationships (connections) that link nodes within a network

Structure: Networked sets of nodes and the ties that connect them

Characteristic: A feature or quality belonging to a node

Indegree/indegree centrality: The number of alters that nominate a given ego

Betweenness centrality: How often an individual falls on the shortest relationship path between two other individuals in the network; reflects the extent to which an individual mediates other relationships

Outdegree/outdegree centrality: The number of people an individual selects/nominates within the network

Reciprocity: Whether social network members mutually nominate each other, can be applied to both indegree and outdegree nominations

Mutuality: the extent to which social network members nominate each other, calculated by dividing the number of reciprocated ties by the total number of unreciprocated ties plus the total number of reciprocated ties.

Ego density: the total number of ties between an ego’s nominations divided by the total number of possible ties between the nominations

Prestige: How many connections an ego has and how many connections the alters of the ego has, and so on

Group integration: The extent to which an ego’s outdegree nominations are in a bounded social network (e.g. a school), including within sub-networks (e.g. grades)

Network density: The total number of observed connections divided by the maximum number of possible connections

Transitivity: The extent to which the relation between two members in a shared social network that are connected by another member is transitive, or put more plainly, that friends of a person’s friends are also his friends

Closeness: The minimum number of ties needed to reach all the other individuals in the network

Gender heterophily: An index of how many cross-gender relationships an ego nominates

Effective size: The number of alters that ego has, minus the average number of ties that each alter has to other alters

Cluster identification: Identifying clusters within a network by progressively deleting the edges with the highest edge betweenness

Homophily: The tendency for members of a shared social network to share similar characteristics

### Objectives

An objective of all the included studies was to assess how a social network characteristic was associated with alcohol consumption. Specifically, 12 studies assessed the association between characteristics of network members (e.g. peers’ weekly alcohol consumption or peers’ beliefs about alcohol) and alcohol consumption [68, 70, 71, 73–76, 78–80, 82, 83]. Eight studies assessed the association between characteristics of network structure, such as network size, network shape, network attributes, or position within a network, and alcohol consumption [67–69, 72, 77, 78, 81, 82].

### Measures

The measures used in the included studies were heterogeneous. Table 3 provides definitions for the social network characteristics that the studies examined, which included: attributes of network members (e.g. peers’ weekly alcohol consumption or peers’ beliefs about alcohol), indegree/indegree centrality, betweenness centrality, outdegree/outdegree centrality, reciprocity, prestige, group integration, density, transitivity, closeness, gender heterophily, effective size, cluster identification, and homophily.

**Table 3:** Social network measures used in the identified studies and their definitions.

Social network measure	Definition
Indegree/indegree centrality	The number of alters that nominate a given ego
Betweenness centrality	How often an individual falls on the shortest relationship path between two other individuals in the network; reflects the extent to which an individual mediates other relationships
Outdegree/outdegree centrality	The number of people an individual selects/nominates within the network
Reciprocity	Whether social network members mutually nominate each other, can be applied to both indegree and outdegree nominations
Prestige	How many connections an ego has and how many connections the alters of the ego has, and so on
Group integration	The extent to which an ego’s outdegree nominations are in a bounded social network (e.g. a school), including within sub-networks (e.g. grades)
Network density	The total number of observed connections divided by the maximum number of possible connections
Transitivity	The extent to which the relation between two members in a shared social network that are connected by another member is transitive, or put more plainly, that friends of a person’s friends are also his friends
Closeness	The minimum number of ties needed to reach all the other individuals in the network
Gender heterophily	An index of how many cross-gender relationships an ego nominates
Effective size	The number of alters that ego has, minus the average number of ties that each alter has to other alters
Cluster identification	Identifying clusters within a network by progressively deleting the edges with the highest edge betweenness
Homophily	The tendency for members of a shared social network to share similar characteristics

Definitions

Nodes: Distinct members of a social network (e.g., study participants)

Ego: An individual focal node providing information about their social network

Alter: The nodes to whom an ego is directly connected

Ties (edges): Representations of relationships (connections) that link nodes within a network

Structure: Networked sets of nodes and the ties that connect them

Characteristic: A feature or quality belonging to a node

All the studies measured some form of alcohol consumption over a specific time period as the outcome. These included frequency of alcohol consumption, quantity of alcohol consumption, and frequency of binge drinking. Some studies used a single item to measure alcohol consumption, while others measured multiple forms of alcohol consumption using previously validated scales. One study looked at frequency of drug and/or alcohol use with a social network member in the past 6 months without distinguishing between drug and alcohol use [72]. The naturalistic observation study relied on observed counts of alcoholic drinks consumed [80], all the other studies relied on self-reported data, including one that compared perceived levels of alcohol consumption by peers to self-reported alcohol consumption by participants [79].

### Statistical analyses

The statistical analyses used to assess social network characteristics were also heterogeneous, although the majority used some form of regression modeling correcting for nonindependence of observations/autocorrelation among network members. The longitudinal studies used Generalized Estimating Equations (GEE) to account for repeated measures. Overall, the methodologies described by the included studies were rigorous enough that the studies met inclusion criteria based on the quality assessment tool (mean = 95%).

### Findings

All the included studies reported a statistically significant association between a social network characteristic and an alcohol-related outcome. The different types of social network members whose alcohol consumption (or in one study, their beliefs about alcohol [76]) was associated with participants’ alcohol consumption, included: peers/friends [69, 70, 74, 76, 78, 80, 82, 83], dorm mates [68, 73, 79], drug network members [75], and parents [76]. In one study, participants’ alcohol consumption was associated with alcohol consumption of relatives’ and friends’ but not that of immediate neighbors or co-workers [82].

Social network members’ drinking behaviors were associated with participants’ drinking behaviors in multiple ways. For example, among first-year university students in Germany, participants’ quantity of drinking was associated with friends’ quantity of drinking but participants’ frequency of drinking was only associated with friends who also identified the participant as a friend [71]. At a medium-sized university in the Northeast US, weekly volume of alcohol consumed among nominated peers was significantly associated with that of participants but alcohol problems (measured using the 24-item Brief-Young Adult Alcohol Consequences Questionnaire [84]) were not [68]. In a subsequent study that sampled the entire first-year class at the same university, participant's binge drinking frequency was positively associated with both perceived and actual binge drinking frequency of important peers, and resistance to peer influence weakened the effect of perceived peer binge drinking on participant binge drinking, but did not interact with actual norms [70]. In a sub-sample of past-month drinkers from the previous study, the number of drinks consumed by your peers on their heaviest drinking occasion was associated with greater drinking quantities on one's own heaviest drinking occasion [78]. Among black men who have sex with men (MSM) in a community setting in South Africa, individuals whose social networks included a higher proportion of hazardous drinkers were more likely to be hazardous drinkers themselves [74]. In the naturalistic observation study, peer group alcohol consumption was the strongest predictor of participants’ alcohol consumption [80]. Data from the Framingham Heart Study was used to show how the impact of peers drinking habits on one’s own drinking behaviors diminished across degrees of separation, i.e. one’s own friends influence drinking more than the friends of one’s friends [82].

A few studies looked at peer effects in social networks by examining homophily [68, 81, 82]. One study showed that quantity of alcohol consumption clustered within nominated peers of a university residence hall, then used regression to show that participants’ quantity of alcohol consumption was associated with nominated peers’ quantity of alcohol consumption [68]. Two studies observed that homophily increased over time (i.e. social networks became more homophilous), and provided support for induction because the directionality of friendship nominations mattered after controlling for participants’ previous alcohol consumption [81, 82].

Certain studies assessed associations between other aspects of social network structure than homophily (sometimes in addition to looking at the association between social network members drinking behaviors and participants’ drinking behaviors), such as network size, network shape, network attributes or position within a network, and alcohol-related outcomes [67, 72, 77]. For example, among university students in the US, outdegree was positively associated with number of heavy drinking days [67]. In the same study, betweenness centrality was positively associated with alcohol-related problems, with a stronger association among women [67]. In a subsequent study that sampled the entire first-year class at the same university, popularity (indegree) was positively associated with participants’ alcohol consumption, binge drinking frequency, and alcohol-related problems [69]. In a sub-sample of past-month drinkers from the previous study, being present at other peers' heaviest drinking occasions was associated with greater drinking quantities on one's own heaviest drinking occasion [78]. Among university students in Belgium, indegree was positively associated with binge drinking, while gender heterophily and effective size were negatively associated [77]. Among young, MSM in a community setting in the US, transitivity was positively associated with frequency of alcohol and/or drug use [72]. Taken together, these findings suggest that characteristics of social networks and one’s position in a social network also are associated with alcohol-related outcomes.

### Critical evaluation

Almost all (15 out of 17) of the included studies were conducted in young adults [67–74, 76–81, 83], mostly in university settings [67–71, 73, 76–81], with just 2 in community settings [72, 74]. Two studies were conducted among adults whose mean age was greater than 30 [75, 82]. Considering the ways that social networks are formed, it is possible that school settings, in which individuals live in close proximity to similar-aged peers who share a large number of commonalities, have a qualitatively different effect on the ways that social networks are formed and how information is transmitted through these networks than networks of older adults or those in community settings.

The variation in measures used, both of social network characteristics and alcohol consumption, makes it challenging to summarize the body of evidence for how social networks influence alcohol consumption in young adults, and vice versa. Also as a limitation, many of the studies were cross-sectional [67–70, 72–74, 77–79, 83]. Cross-sectional designs allow researchers to test for correlations between an individuals’ alcohol consumption and that of their peers, but not to distinguish whether the correlations result from selection, confounding or induction (i.e. that the observed homophily with regards to alcohol consumption observed among social networks of adults is due to the tendency for people to befriend those similar to themselves, the effects of shared environments, or the spread of drinking behaviors within networks). Two studies examined directionality and found support for induction [81, 82].

Some studies collected data on complete networks [77, 81] or used complete lists of members in a social network (i.e. rosters) but limited the number of peers that participants were able to nominate [67–71, 73, 78, 79]. Other studies relied on peer nominations or recruitment without the availability of rosters [72, 74–76, 80, 82, 83]. The studies with less complete networks suffer from missing data that raise doubt about whether the results apply to the entire network, and whether the network metrics are accurate. Dyadic index-peer data can provide useful information, but is incomplete social network data because it limits researchers to investigation of the effects of peer consumption rather than social network position or other characteristics of social network structure. Such studies contribute only limited information about how social networks influence adult alcohol use. Furthermore, results that rely on incomplete social network data are likely to suffer from selection bias because peers who are closer to the participant (and thus, more likely to share similar characteristics) are more likely to be selected into the study. While many of the studies in adults were limited in the completeness of social network data collected; collectively, they featured a wide variety of types of social network members studied (e.g. peers/friends, classmates/dormmates, drug network members, relatives/parents, neighbors, co-workers). In a few of the studies [72, 74, 83], the nature of the relationships among participants was not even known, just that there was some sort of connection between them because one participant’s recruitment was attributed to the other. This is reflective of how peer groups are more expansive and diverse in adulthood [60], but also more difficult to capture completely. In nearly all cases, these social network members were found to influence participant alcohol consumption. Lastly, many of the included studies were conducted among specific populations (e.g. fraternity brothers [81], injection drug users [75], men who have sex with men [72, 74]), raising questions about the generalizability of the findings.

## Discussion

Through a systematic review, 17 studies were identified and evaluated to assess the evidence on whether social network characteristics were associated with drinking behaviors in adults. These studies measured and analyzed social networks in various ways. The heterogeneity of methods used make it difficult to generalize about how, specifically, social networks influence alcohol consumption and vice versa. However, in young adults, among whom the majority of studies were conducted, with whom they socialize and how they socialize appears to be associated with alcohol consumption across methodologies and settings.

This review identified a lack of research on social network characteristics and alcohol consumption among middle-aged or older adults. Therefore, there is limited evidence to infer how social networks are associated with alcohol consumption further into adulthood, although it is likely quite different for a number of reasons. First, social network dynamics differ throughout the lifespan [60]. For example, as adolescents enter into young adulthood, their social networks tend to grow in size, and the strength of peer dynamics remain strong, especially when young adults are located in settings with relatively complete (i.e. bounded) social networks, such as universities [62]. The influence of peers is believed to wane in later adulthood [62]. Second, drinking patterns also change during the life course [2, 85]. For example, entering college [86] or meeting the legal minimal age to purchase alcohol are known to alter drinking behaviors and alcohol-related harms [87]. Third, alcohol use also has changing physiological effects as individuals age [88], with increasing health risks associated with aging [89–91]. Whether sociometric social network data would be particularly informative for examining alcohol use in older adults remains an open question, especially given that network effects might be waning [62]. Sorting out these questions remains an area that requires further research. Another important area that was not explored in the literature that we identified is a comparison of the impact of social networks on alcohol use between adults and adolescents. Using a life course perspective to explore the changing impacts of social networks on alcohol use could be further enlightening.

An important question is whether social network analysis-based peer effects on drinking are different in adults than in adolescents. No reviewed studies directly investigated this. One study [92] investigated the effects of adolescent social network characteristics on participants in the National Longitudinal Study of Adolescent to Adult Health (Add Health) [58] when they were adolescents and then re-examined the effects of the adolescent social network characteristics on participants when they had become young adults. One characteristic, adolescent group integration, showed a weaker effect on binge drinking in adulthood than adolescence, while another adolescent social network characteristic, prestige, showed an increased effect on adult binge drinking, suggesting that the lasting effects of adolescent social network analysis-based peer effects into adulthood varied depending on the characteristic considered. However, this study was not included in the main review because it did not meet inclusion criteria (the social network recruitment was done during adolescence, not adulthood). Comparing how the studies in the current review on adults differed from the studies in the previous review that included studies of adolescents [57]: in adolescents, the social network data collected was much more complete and many social network characteristics were able to be explored, yet peer effects were almost exclusively examined in schoolmates, with studies distinguishing further about friendship, closeness, reciprocity, and shared affiliations (e.g., sports or club activities). The relationships studied in the social network literature among adults were more extensive, and included peers/friends, classmates/dormmates, drug network members, relatives/parents, neighbors, co-workers. While having more expansive and diverse social networks as adults makes collecting complete social network data more costly and challenging, one of the benefits of collecting this information is that they will help us more fully understand how social networks characteristics affect alcohol consumption among adults.

Social network research methods are still relatively novel for studying alcohol use in adults, with 14 of the 17 included papers published since 2010. This literature is likely to continue growing as social network research is expanding [50, 93], and methodologies are being refined [13]. This review demonstrates the adaptability of social network analysis to study alcohol consumption, which is a prime topic for social network research as drinking (unlike many other health-related behaviors) is often undertaken as a shared social activity. The social aspects of drinking are especially evident in teens and young adults, but not as clear as people get older. Hopefully, as this field grows, more studies will be conducted among older adults and in community settings, where sociometric network data is more challenging to collect, but potentially more informative.

This review and the study results have several limitations. First, only 17 studies were identified and deemed eligible to be included based on relevance and quality. Articles may have been missed because the use of social network methods was not indicated in the title or abstract and thus they would not have been identified during the literature search. However, for that reason, a broad search was conducted and ultimately a large number (5,510) of articles were screened based on title and abstract. Second, all studies included in the review reported at least one statistically significant association; this might be a reflection of publication bias. Third, the results of social network analysis studies are context-specific, and insights are likely to vary based on setting and the exposures and the outcomes that were measured. Furthermore, even findings within studies varied by whether the outcome was frequency of alcohol consumption, quantity of alcohol consumption, or binge drinking. It might be important to consider implementing some level of standardization across social network studies, and to prioritize assessing the replicability of findings in different settings.

Despite the limitations, this review identified numerous studies that have applied social network analysis to study alcohol consumption in (mostly young) adult populations. Social network analysis is a method that helps us better understand alcohol use in young adults because it not only addresses the actual drinking behaviors of their peers, but also uniquely addresses how various characteristics of social network structure (e.g. homophily, popularity, transitivity) are associated with individual alcohol use. In other words, how young adults socialize and who they socialize with appears to matter when alcohol consumption is considered. In this sense, social network analysis is a useful tool with the potential to explore the effects of social mixing patterns on alcohol consumption.

### Implications for research and intervention

There are a few important take-away messages from this first review of the growing number of empirical studies that have used social network analysis to explore alcohol use in adults. First, peer alcohol use and other social network characteristics (e.g. network attributes or position within a network) were associated with adults’ alcohol consumption across studies. Second, we identified a lack of research on the impact of social networks on alcohol use in middle-age and older adults, especially those residing in community settings (i.e. without explicit boundaries). Future empirical research should work to address these gaps in our understanding. Efforts should also be made to reduce heterogeneity in social network analysis studies (e.g. agreeing on standardized definitions of social network measures, assessing and reporting the results of them consistently) to facilitate generalizability. This review informs alcohol researchers, health service providers, and policymakers about how social networks have been studied, thus far, to better understand alcohol consumption. As social networks appear to play a role in the consumption of alcohol in young adulthood, this suggests potential for interventions that utilize social networks to help reduce the burden of harmful alcohol consumption [92]. Interventions that utilize social networks to promote behavior change are increasingly available [44], calling for studies of their feasibility and efficacy in reducing alcohol consumption in young adult populations.

## Supporting information

S1 Table PRISMA 2009 checklist. (DOCX)

S2 Table Literature review database search strategy and terms. (DOCX)

S3 Table Quality assessment tool. (DOCX)

S4 Table Quality assessment of articles selected for review. (DOCX)
