# Supplementary material for: Using social network analysis to examine alcohol use among adults: A systematic review
Source: PLoS One. 2019 Aug 22;14(8):e0221360. doi: 10.1371/journal.pone.0221360 (PMC6705782; doi:10.1371/journal.pone.0221360)
Supplement: S2 Table — (DOCX) [file pone.0221360.s002.docx]

**S2 Table** Literature review database search strategy and terms

| **Database** | **Search strategy and terms** | **Records identified** |
| --- | --- | --- |
| PubMed/MEDLINE | (((((((("alcohol use" OR "alcohol user" OR "alcohol users" OR "alcohol misuse" OR "alcohol abuse" OR "alcohol abuser" OR "alcohol abusers" OR "alcohol addict" OR "alcohol addicts" OR "alcohol dependence" OR "alcohol dependent")) OR ("drinker" OR "drinkers" OR "drinking" OR "alcoholic" OR "alcoholics")) OR "Alcohol-Related Disorders" [mh:noexp]) OR "Alcoholic Intoxication" [mh:noexp]) OR "Alcoholism" [mh:noexp]) OR "Binge Drinking" [mh:noexp])) AND (("Social Support"[Mesh]) OR (((((((("social network") OR "social networks") OR "network analysis") OR "network analyses") OR "friendship network") OR "friendship networks") OR "peer networks") OR "peer network")) | 2234 |
| EMBASE | ("alcohol use" or "alcohol user" or "alcohol users" or "alcohol misuse" or "alcohol abuse" or "alcohol abuser" or "alcohol abusers" or "alcohol addict" or "alcohol addicts" or "alcohol dependence" or "alcohol dependent").mp. [mp=title, abstract, heading word, drug trade name, original title, device manufacturer, drug manufacturer, device trade name, keyword, floating subheading word]  Or  ("drinker" or "drinkers" or "drinking" or "alcoholic" or "alcoholics").mp. [mp=title, abstract, heading word, drug trade name, original title, device manufacturer, drug manufacturer, device trade name, keyword, floating subheading word]  Or  alcoholism/ or alcohol abuse/  And  ("social network" or "social networks" or "network analysis" or "network analyses" or "friendship network" or "friendship networks" or "peer networks" or "peer network").mp. [mp=title, abstract, heading word, drug trade name, original title, device manufacturer, drug manufacturer, device trade name, keyword, floating subheading word]  Or  Social network/  Or  Social support/ | 2235 |
| PsycINFO | ("alcohol use" or "alcohol user" or "alcohol users" or "alcohol misuse" or "alcohol abuse" or "alcohol abuser" or "alcohol abusers" or "alcohol addict" or "alcohol addicts" or "alcohol dependence" or "alcohol dependent").mp. [mp=title, abstract, heading word, drug trade name, original title, device manufacturer, drug manufacturer, device trade name, keyword, floating subheading word]  Or  ("drinker" or "drinkers" or "drinking" or "alcoholic" or "alcoholics").mp. [mp=title, abstract, heading word, drug trade name, original title, device manufacturer, drug manufacturer, device trade name, keyword, floating subheading word]  Or  alcoholism/ or alcohol abuse/  And  ("social network" or "social networks" or "network analysis" or "network analyses" or "friendship network" or "friendship networks" or "peer networks" or "peer network").mp. [mp=title, abstract, heading word, drug trade name, original title, device manufacturer, drug manufacturer, device trade name, keyword, floating subheading word]  Or  Social networks/ or social support/ | 950 |
| Web of Science | ("alcohol use" OR "alcohol user" OR "alcohol users" OR "alcohol misuse" OR "alcohol abuse" OR "alcohol abuser" OR "alcohol abusers" OR "alcohol addict" OR "alcohol addicts" OR "alcohol dependence" OR "alcohol dependent" OR "drinker" OR "drinkers" OR "drinking" OR "alcoholic" OR "alcoholics") AND TOPIC: ("social network" OR "social networks" OR "network analysis" OR "network analyses" OR "friendship network" OR "friendship networks" OR "peer networks" OR "peer network") | 488 |
