# Supplementary material for: Using social network analysis to examine alcohol use among adults: A systematic review
Source: PLoS One. 2019 Aug 22;14(8):e0221360. doi: 10.1371/journal.pone.0221360 (PMC6705782; doi:10.1371/journal.pone.0221360)
Supplement: S3 Table — (DOCX) [file pone.0221360.s003.docx]

**S3 Table.** Quality assessment tool

| 1. Is the research question clear and adequately substantiated? 0 = Inadequately described 1 = Adequately described 2 = Very clear and well substantiated |
| --- |
| 2. Does the study include dates and sources for data collection? 0 = No 1 = Yes |
| 3. Is the description of the study setting adequate? 0 = No 1 = Yes |
| 4. Adequate sample size, where applicable  0 = No or cannot be determined 1 = Yes  Not applicable |
| 5. Adequate response rate (>60%), where applicable 0 = No or cannot be determined 1 = Yes  Not applicable |
| 6. Adequate sample selection 0 = No or cannot be determined 1 = Yes |
| 7. Exposure measurement: Does the study clearly describe collection of social network data (i.e., sociometric interviews)? 0 = No description 1 = Mentioned, little description 2 = Detailed description |
| 8. Outcome measurement: Does the study clearly describe the outcome measure? 0 = No description of case definition 1 = Self-reported by participant 2 = Used a validated measure or provided valid justification for not doing so |
| 9. Social network analysis 0 = No social network measures calculated 1 = Social network measures calculated but not correlated with outcome 2 = Social network measures calculated and correlated with outcome and/or used to define comparison groups |
| 10. Is the description of findings thorough and are the data presented adequately? 0 = No 1 = Adequate 2 = Very thorough |
| 11. Are the strengths and limitations adequately considered? 0 = No 1 = Yes |
| 12. Are the study conclusions supported by the results? 0 = No 1 = Possibly 2 = Yes |
