# Supplementary material for: Using social network analysis to examine alcohol use among adults: A systematic review
Source: PLoS One. 2019 Aug 22;14(8):e0221360. doi: 10.1371/journal.pone.0221360 (PMC6705782; doi:10.1371/journal.pone.0221360)
Supplement: S4 Table — (DOCX) [file pone.0221360.s004.docx]

**S4 Table.** Quality assessment of articles selected for review

| **Article** | **1.**  **Research question** | **2.**  **Data collection** | **3.**  **Study setting** | **4.**  **Sample size** | **5.**  **Response rate** | **6.**  **Sample selection** | **7.**  **Exposure measurement** | **8.**  **Outcome measurement** | **9.**  **Social network analysis** | **10.**  **Findings and data presentation** | **11.**  **Strengths and limitations** | **12.**  **Conclusions** | **Quality score** |
| --- | --- | --- | --- | --- | --- | --- | --- | --- | --- | --- | --- | --- | --- |
| Barnett et al. (2014a) | 2 | 1 | 1 | 1 | 1 | 1 | 2 | 2 | 2 | 2 | 1 | 2 | 100% |
| Barnett et al. (2014b) | 1 | 1 | 1 | 1 | 1 | 1 | 1 | 2 | 2 | 1 | 1 | 2 | 83% |
| DiGuiseppi et al. (2018a) | 1 | 1 | 1 | 1 | 1 | 1 | 2 | 2 | 2 | 2 | 1 | 2 | 100% |
| DiGuiseppi et al. (2018b) | 1 | 1 | 1 | 1 | 1 | 1 | 2 | 2 | 2 | 2 | 1 | 2 | 100% |
| Giese et al. (2017) | 2 | 1 | 1 | 1 | 1 | 1 | 2 | 2 | 2 | 1 | 1 | 2 | 94% |
| Janulis et al. (2015) | 2 | 1 | 1 | 1 | 1 | 1 | 2 | 1 | 2 | 1 | 1 | 2 | 89% |
| Kenney et al. (2017) | 2 | 1 | 1 | 1 | 1 | 1 | 2 | 2 | 2 | 2 | 1 | 2 | 100% |
| Knox et al. (2017) | 2 | 1 | 1 | 1 | 0 | 1 | 2 | 2 | 2 | 2 | 1 | 2 | 94% |
| Latkin et al. (1996) | 2 | 1 | 1 | 1 | 1 | 1 | 2 | 1 | 2 | 2 | 1 | 2 | 94% |
| Lau et al. (1990) | 1 | 1 | 1 | 1 | 1 | 1 | 2 | 2 | 2 | 1 | 1 | 1 | 89% |
| Lorant et al. (2015) | 2 | 1 | 1 | 1 | 1 | 1 | 2 | 2 | 2 | 2 | 1 | 2 | 100% |
| Meisel et al. (2018) | 1 | 1 | 1 | 1 | 1 | 1 | 2 | 2 | 2 | 2 | 1 | 2 | 100% |
| Ott et al. (2016) | 2 | 1 | 1 | 1 | 1 | 1 | 2 | 2 | 2 | 2 | 1 | 2 | 100% |
| Overbeek et al. (2010) | 2 | 1 | 1 | 1 | 1 | 1 | 2 | 2 | 2 | 2 | 1 | 2 | 100% |
| Phua (2011) | 1 | 1 | 1 | 1 | 1 | 1 | 2 | 1 | 2 | 1 | 1 | 1 | 78% |
| Rosenquist et al. (2010) | 2 | 1 | 1 | 1 | 1 | 1 | 2 | 2 | 2 | 2 | 1 | 2 | 100% |
| Tucker et al. (2015) | 2 | 1 | 1 | 1 | 1 | 1 | 2 | 2 | 1 | 2 | 1 | 2 | 94% |
